# Arrhythmia insensitive rapid cardiac T1 mapping pulse sequence

**DOI:** 10.1186/1532-429X-15-S1-O112

**Published:** 2013-01-30

**Authors:** Michelle Fitts, Elodie Breton, Eugene Kholmovski, Derek J Dosdall, Sathya Vijayakumar, Kyung P Hong, Ravi Ranjan, Nassir F Marrouche, Leon Axel, Daniel Kim

**Affiliations:** 1 Radiology, University of Utah, Salt Lake City, UT, USA; 2 CARMA, University of Utah, Salt Lake City, UT, USA; 3 Bioengineering, University of Utah, Salt Lake City, UT, USA; 4 Internal Medicine, University of Utah, Salt Lake City, UT, USA; 5 Equipe Automatique, Vision et Robotique, Laboratoire des Sciences des Sciences de l'Image, de l'Informatique et de la Télédétection, Université de Strasbourg, Strasbourg, France; 6 Radiology, New York University, New York, NY, USA

## Background

Cardiac fibrosis is a known marker of adverse remodeling of the heart. Late-gadolinium-enhanced (LGE) T1 mapping is the only proven method to assess diffuse myocardial fibrosis. The most widely used LGE cardiac T1 mapping pulse sequence is MOLLI, which is based on inversion-recovery (IR) magnetization pre-conditioning and Look-Locker imaging. Unfortunately, MOLLI is sensitive to heart rate and rhythm and T2 effects and requires a long breath hold duration. We present an arrhythmia-insensitive, rapid (AIR) cardiac T1 mapping pulse sequence which is also insensitive to T2 effects.

## Methods

We developed the AIR cardiac T1 mapping pulse sequence based on B1-insensitive saturation recovery (SR) magnetization pre-conditioning (insensitive to heart rate-rhythm) and two single-shot balanced steady-state free precession (b-SSFP) image acquisitions (proton density and T1-weighted) with centric k-space ordering (rapid, insensitive to T2 effects). We compared its performance against MOLLI in an arrhythmia phantom (T1 ranging from 500 - 2000 ms; 10 repetitions to assess repeatability) with an effective heart rate of 111 beat-per-minute (bpm) and in ten human subjects and 17 large animals in sinus rhythm pre-contrast and 5, 10, and 15 min post contrast agent (0.1 mmol/kg of Gd-BOPTA) administration.

## Results

**Figure 1:**
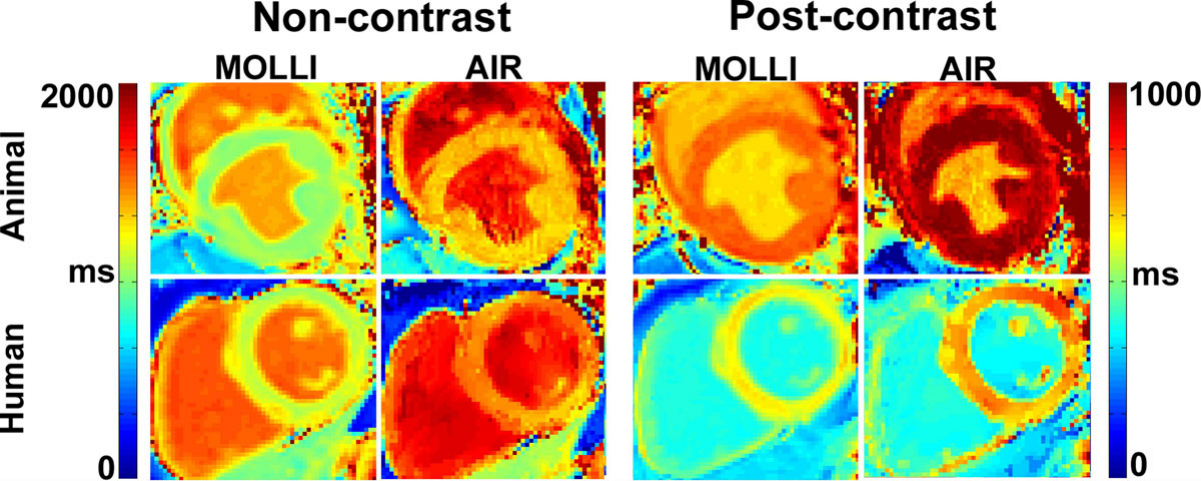
Non-contrast and post-contrast MOLLI and AIR cardiac T1 maps for a baseline (prior to pacing) dog and a 38-year-old male volunteer. Compared with AIR, MOLLI yielded lower T1 values but more T2 blurring. T1 map quality is comparable between the two methods.

**Figure 2:**
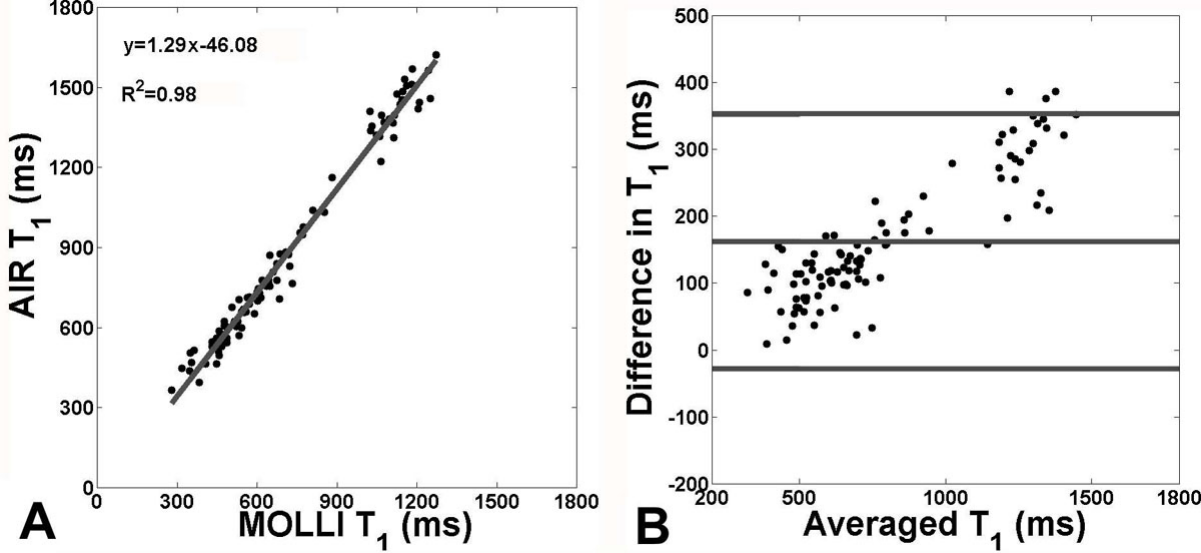
A) Pearson's correlation and B) Bland-Altman analyses results for MOLLI and AIR T1 measurements *in vivo*.

Compared with the reference T1 measured by IR fast spin-echo pulse sequence in the phantom, MOLLI and AIR T1 measurements had maximum coefficient of variation (CV) of 10% and 0.8%, respectively, at arrhythmia and normalized root-mean-square-error (NRMSE) of 22% and 3%, respectively, at arrhythmia. For in vivo, mean heart rates in humans, dogs, and goats were 57±9 bpm, 86±15 bpm, and 107±15 bpm, respectively. Compared with AIR T1 maps, MOLLI T1 maps yielded lower values but more T2 blurring (Fig.1). As shown in Figure 2, T1 measurements made by MOLLI and AIR were strongly correlated (r=0.99) but in poor agreement 161.77ms, upper and lower 95% limits of agreements = 347.54ms and -24.01ms). Averaging results over 10 humans, compared with MOLLI T1 (1198±46 ms), AIR T1 measurement (1501 ± 69 ms) agreed better with a previous study which measured T1 (1471 ms) of an excised heart using a rigorous IR pulse sequence with 35 TI values with 2 averages.

## Conclusions

Our findings in vitro and in vivo suggest that AIR is more accurate than MOLLI for cardiac T1 mapping. This rapid cardiac T1 mapping pulse sequence may be clinically useful for assessment of diffuse myocardial fibrosis in patients with rapid heart rates and/or in arrhythmia.

## Funding

American Heart Association: 0730143N; Ben B. and Iris M. Margolis Foundation.

